# Complex Visual Adaptations in Squid for Specific Tasks in Different Environments

**DOI:** 10.3389/fphys.2017.00105

**Published:** 2017-02-24

**Authors:** Wen-Sung Chung, N. Justin Marshall

**Affiliations:** Sensory Neurobiology Group, Queensland Brain Institute, The University of QueenslandSt Lucia, QLD, Australia

**Keywords:** magnetic resonance imagery, retinal deformation, dual-layered inner segment, complex squid retina, mid-water, optic lobe, signal convergence

## Abstract

In common with their major competitors, the fish, squid are fast moving visual predators that live over a great range of depths in the ocean. Both squid and fish show a variety of adaptations with respect to optical properties, receptors and their underlying neural circuits, and these adaptations are often linked to the light conditions of their specific niche. In contrast to the extensive investigations of adaptive strategies in fish, vision in response to the varying quantity and quality of available light, our knowledge of visual adaptations in squid remains sparse. This study therefore undertook a comparative study of visual adaptations and capabilities in a number of squid species collected between 0 and 1,200 m. Histology, magnetic resonance imagery (MRI), and depth distributions were used to compare brains, eyes, and visual capabilities, revealing that the squid eye designs reflect the lifestyle and the versatility of neural architecture in its visual system. Tubular eyes and two types of regional retinal deformation were identified and these eye modifications are strongly associated with specific directional visual tasks. In addition, a combination of conventional and immuno-histology demonstrated a new form of a complex retina possessing two inner segment layers in two mid-water squid species which they rhythmically move across a broad range of depths (50–1,000 m). In contrast to their relatives with the regular single-layered inner segment retina live in the upper mesopelagic layer (50–400 m), the new form of retinal interneuronal layers suggests that the visual sensitivity of these two long distance vertical migrants may increase in response to dimmer environments.

## Introduction

**Figure 1:**
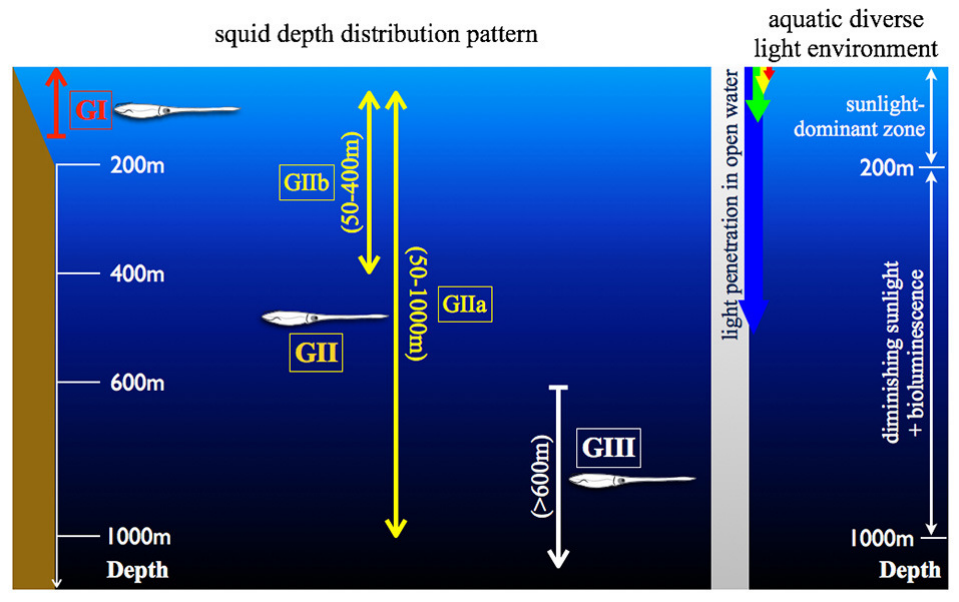
**Illustration of the diverse aquatic light condition and the squid depth distribution**. (GI) indicates the coastal squid, including *Idiosepius notoides* and *Sepioteuthis lessoniana*. (GII) as the squid possess daily vertical migration and can be further divided into two subgroups (GIIa and GIIb) depending on the migration depth range. GIIa includes *Abraliopsis falco, Liocranchia reinhartdi* and *Spirula spirula*; GIIb: *Pyrotuethis margaritifera*. (GIII) as the squid constantly inhabit in the scotopic environment, including *Bathyteuthis abyssicola*. These categorized groups are consistently used in Figures 4, 7, 8.

Fish and squid are both successful visual predators. Having high sensitivity is one requirement for visual predators in foraging under the low light conditions and for detecting fast-moving objects. The light intensity in the aquatic world is largely determined by two factors, time of day (availability of sunlight) and depth (scattered and absorbed by waters) (Denton, 1990; Johnsen, 2012). After dusk, the light level at the surface drops by 8 log units compared to mid-day. Another important feature of underwater light condition is that the intensity of the downwelling sunlight is depth-dependent, with a 10-fold drop in brightness with every 75 m depth increase, even in clear open ocean (Denton, 1990). In addition, the spectral range is gradually tuned to nearly constant blue spectra over increasing depths and in clear ocean to around 475 nm (Figure 1). In more coastal waters, this is green-shifted (Jerlov, 1976; Lythgoe, 1979). These diverse aquatic photonic environments have driven a variety of visual adaptations across different fauna (Lythgoe, 1979; Warrant and Locket, 2004; Yokoyama, 2008; Cronin et al., 2014; Chung and Marshall, 2016).

Biodiversity and fishery surveys show that squid occur over a great range of depths similar to fish (Marshall, 1979; Jereb and Roper, 2005, 2010). Their depth distribution patterns can be categorized into three major groups (Figure 1): (1) Coastal group (GI) inhabiting between 0 and 200 m depth. (2) Pelagic group (GII) inhabiting the water column with diurnal vertical migration between surface and hundreds of meters. (3) Deep pelagic group (GIII) inhabiting permanently scotopic depths (Clarke and Lu, 1974, 1975; Lu and Clarke, 1975a,b). Accumulated videography has confirmed that many mid-water squid are capable of reacting to point-like light as well as prey-predator interactions under similar levels of brightness as other inhabitants (Kubodera et al., 2007; Bush et al., 2009; Gilly et al., 2012). Squid are attractive for studying the evolution of vision as they have camera-like eyes sharing optical, anatomical and functional characteristics with fish, while having evolved these parallels through convergence (Packard, 1972). It is perhaps not surprising that most of these comparative studies focus on easy-to-access coastal squid, with the visual adaptation of deep-sea squid remaining poorly studied (Sivak, 1991; Sweeney et al., 2007; Makino and Miyazaki, 2010). Our goal in this study was therefore to show the various and complex adaptations in the morphology and the underlying circuitry of the squid visual system using a number of squid collected between 0 and 1,200 m.

In the mesopelagic environment (200–1,000 m depth), food and mates are not abundant and decreasing visibility through light attenuation results in strong selection pressures for remarkable visual adaptation (Warrant and Locket, 2004; Nilsson et al., 2012). In order to live in dim environments, fishes have developed many adaptations to improve sensitivity using optical improvements (i.e., spectral tuning, multi-banked rod retina, tapetum, diverticular, and tubular eyes) and neural summation or convergence (Lythgoe, 1979; Wagner et al., 1998; Warrant and Locket, 2004; Yokoyama, 2008; Partridge et al., 2014). Squid have successfully adapted to diverse aquatic visual environments, though different species might have adapted in different ways depending on the habitat light conditions. Deep-sea squid show a remarkable diversity of eye design as first noted by Chun (1910), however this has rarely been linked to photonic condition (Land, 1981). Aside from occasional reports of retinal adaptation (i.e., the fovea of *Bathyteuthis*; the dimorphic eyes of *Histioteuthis*; the elongated banked photoreceptors of *Watasenia*), the main body of knowledge in deep-sea squid visual performance is restricted to optical properties using comparisons of gross anatomy of eyes and optical qualities of the lens (Young, 1972, 1975a; Sivak, 1991; Land, 1992; Michinomae et al., 1994; Sweeney et al., 2007).

In contrast to remarkable adaptations of squid eye in morphology and optics, squid visual adaptations, particularly at the cellular and neural level, are rarely explored (Chun, 1910; Young, 1963; Sweeney et al., 2007; Makino and Miyazaki, 2010). Many previous studies revealed that squid possess a structurally simple retina, comprised of a single receptor layer and a single retinal plexus layer (Cajal, 1917; Cohen, 1973; Daw and Pearlman, 1974). The main function of the photosensitive rhabdomeric layer of the retina was thought to be photon absorption alone and thus, investigations of squid visual adaptations inside the retina have also been largely ignored (Cajal, 1917; Cohen, 1973). To date, a large portion of studies of squid visual system and the associating neural network relies on classical serial histological sectioning (Maddock and Young, 1987; Wild et al., 2015). The methodological constraints of classical histology to a single angle per specimen is clearly a limiting factor, particularly in rare deep-sea species. In order to overcome this, firstly, a contrast-enhanced magnetic resonance imagery (MRI) protocol was developed to explore the gross morphology of eyes and brains, and retinal topography in seven squid species from different habitats. With the reconstruction of three-dimension MR imagery, we discovered that variable enlargement of eyes and optic lobes, and three newly described types of retinal deformation are associated with different habitats and habits.

Follow-up histological examination found that the enlarged eyes combined with loss of light filtering screening pigments also link to dim light conditions. Furthermore, histological and immunohistological evidence showed that two mid-water squid species known to show regular migratory behavior between 50 and 1,000 m possess a new type of retinal feature in the inner segment layer. Here instead of a single cell layer, two types of retinal cells are found with complex neural interconnections. This new form of the dual-layered inner segment squid retina is suggested to be equivalent to the neural summation mechanism of the vertebrate's retina, improving visual sensitivity and dynamic range of light reception.

## Materials and methods

### Animals

Cephalopods used in this study were collected from surface to 1,200 m depth. Two coastal squid species were collected using a seine net (water depth 1–3 m) close to Moreton Bay Research Station, Stradbroke Island, Queensland. Pelagic cephalopods were sampled using a Rectangular Midwater Trawl Net (RMT) with the trawling speed 0.8–2 knots from four deep-sea cruises. Collecting location and depth range of selected animals are listed in Table 1 and Supplementary Figure 1.

**Table 1:** **List of specimens and their living depth range**.

**Habitats**	**Species**	**Specimens and the associating collection depth range in this study**		**MRI [samples and mantle length (mm)]**	**Histology [samples and mantle length (mm)]**	**Immuno-histology [samples and mantle length (mm)]**	**Notes (sampling methods and locations, and known distribution depths)**
		**Day**	**Night**				
Coastal waters	*Idiosepius notoides*	10 (1–3 m)	5 (1 m)	8 (ML8-14)	6 (ML8-14)	3 (ML10-14)	I^A^, 0–10 m^α^
	*Sepioteuthis lessoniana*	5 (1–3 m)	5 (1 m)	4 (ML13-30)	4 (ML15-45)	3 (ML20-25)	I^A^, 0–100 m^β^
Mid-waters	*Abraliopsis falco*	15 (400–1000 m)	n/a	1 (ML16)	4 (ML16-31)	3 (ML16-24)	III^C^, 400–1,000 m^γ^
	*Pyroteuthis margaritifera*	8 (300–400 m)	26 (50–100 m)	1 (ML12)	4 (ML12-24)	2 (ML14-16)	II^B^^,^^D^^,^^E^, 50–500 m^β^
	*Spirula spirula*	n/a	5 (150–600 m)	2 (ML13, 42)	4 (ML13-42)	–	II^B^^,^^D^^,^^E^, 300–1,750 m^β^
	*Liocranchia reinhardti*	7 (400–1,000 m)	18 (50–100 m)	2 (ML12, 32)	4 (ML25-95)	1 (ML115)	II,III^B^^−^^E^, 0–1,200 m^β^
	*Bathyteuthis abyssicola*	2 (600–1,200 m)	1 (800 m)	2 (ML15, 64)	2 (ML15, 64)	–	II^B^, 700–2,500 m^β^

*Sampling methods: I, Seine net; II, RMT8; III, RMT16*.

Sampling locations and Vessels:

A *, Moreton Bay, Queensland 2010)*;

B *, Coral Sea (RV Cape Ferguson, December 2009)*;

C *, Peru-Chilean Waters (RV Sonne, August 2010)*;

D *, Coral Sea (RV Cape Ferguson, December 2010)*;

E *, Coral Sea (RV Cape Ferguson, May 2011)*.

References of squid living depths:

α *as Jereb and Roper (2005)*;

β *Jereb and Roper (2010)*;

γ *as the current study*.

### Magnetic resonance imagery (MRI) and anatomic examination

Chung and Marshall (2014) developed the contrast-enhanced MRI protocol for a coastal squid, revealing advantages in examining central nervous system, rapid gross anatomical and optical analyses in this soft-bodied creature. Although some previous MRI work showed good results using live *Aplysia* and crayfish (Ziegler et al., 2011), keeping an anesthetized squid alive and still for a long MRI scan still encounters significant difficulties. In an effort to achieve high resolution MRI of squid brain (30 μm voxel resolution), with a few modifications of contrast agent treatment, we expanded the cephalopod MRI examination from the freshly-dead coastal squid to preserved deep-sea squid also. The two coastal species and five mid-water squid species were anesthetized in cold seawater mixed with 2% MgCl_2_ and preserved in neutral formalin in the field and transported back to the laboratory. The freshly-preserved specimens (in neutral formalin less than 2 months after catching) were removed from storage and rinsed repeatedly with 0.1 M PBS to minimize the residue of the fixative. Secondly, four aged-preserved specimens (over a year in 70% EtOH after catching, including 2 *Bathyteuthis abyssicola* and 2 *Spirula spirula*) were removed from the storage and rehydrated through a series of reduced alcohols. Finally, all these preserved samples were soaked into 0.1 M PBS added with MRI contrast agent, 1% ionic Gd-DTPA (Magnevist, Bayer, Leverkusen, Germany), overnight before imaging.

The contrast-enhanced specimen was placed into the fomblin-filled (Fomblin oil, Y06/6 grade, Solvay, USA) container to prevent dehydration and then vacuumed for 15 min to remove air bubbles trapped inside animal body. The container was then placed in a custom-built surface acoustic wave coil (4–25 mm diameter) (M2M Imaging, Brisbane, Australia). Imaging was performed at temperature of 22 ± 0.1°C on a 700 MHz wide-bore microimaging system (Bruker Biospin, Karlsruhe, Germany) consisting of a 16.4 T vertical bore magnet interfaced to an AVANCE II spectrometer (Bruker Biospin, Karlsruhe, Germany) running the imaging software Paravision 4 (Bruker Biospin, Karlsruhe, Germany) in the Centre for Advanced Imaging at the University of Queensland. All scans were performed overnight (12–18 h) using a ${\text{T}}_{2}^{*}$-weighted 3D-Flash sequence (TR/TE = 50 ms/14 ms, average = 8), resulted in voxel resolution between 9 and 30 μm. The individual which obtained the highest voxel resolution in each species was selected for further morphologic and quantitative analysis of eyes and brain lobes.

A series of MR image stacks (Unix files) were imported into the image processing software OsiriX (Version 4.1.2, Pixmeo, Switzerland) for inspection of anatomical structure, post-construction of 3D virtual images and volumetric estimates of lobes and eyes. First, the retinal topography of each species was constructed by measuring the length of receptors per 100 × 100 μm^2^ retinal patch across an entire eye. Identification of brain lobes was based on the published anatomical studies that also aid determining the boundaries between tissue types (Young, 1974, 1976, 1977; Messenger, 1979; Young, 1979; Nixon and Young, 2003; Wild et al., 2015; Koizumi et al., 2016). A region of interest (ROI) was manually segmented and assigned to different ROI-series files using OsiriX. The segmented structure was then used to obtain the quantitative volume using the analysis tool *ROI Volume* in OsiriX. In order to compare the enlargement of eyes and optic lobes across squid species, the volume of the ROI was expressed as a percentage of the total head volume.

### Histology of the deformed squid retinal structure

When regionally differentiated eye structure and the corresponding visual axis region were confirmed by MRI, image-guided information of the deformed retinal structure was used to decide the best sectioning angle for light microscopy (as the red dash line shown in **Figure 4**). The retinal sample was repeatedly rinsed with 0.1 M PBS to remove the fomblin oil and then transferred into cryoprotectant (30% sucrose mixed with 0.1 M PBS) prior to embedding in the mounting medium, Optimal Cutting Temperature compound (OCT) (Tissue-Tek, Sakura Finetek, USA) mixed with 10% sucrose. The eyecup was cut at 12 μm thickness at −25°C using a cryostat (CM1100, Leica, Germany) and stained in Haematoxylin and Eosin.

### Histology of the light- and dark-adapted squid eyes

In an effort to study dynamic screening pigment movement, all living specimens were separated into two light-treated groups where one group was exposed to the room light and the other one was kept in a lightproof tank for 1 h dark-adaptation before fixation. The light-adapted animals were deeply anesthetized in 2% MgCl_2_ mixed seawater and then decapitated and fixed in 4% neutral paraformaldehyde (PFA) mixed seawater. The dark-adapted specimens were anesthetized and decapitated under dim red illumination, and kept in the lightproof containers with 4% PFA until sectioning. The retinal segments were rinsed repeatedly with 0.1M PBS and used with tangential section by a standard cryosectioning procedure and H&E staining. Lengths of the rhabdom, dynamic movements of screening pigment granules were imaged using a Zeiss microscope (Axioscop- HBO 50) and measured using the software Fiji (NIH, USA).

### Estimates of photoreceptor density at the visual axis region

Young (1963) described the “simple cephalopod retina” where the major function of the photoreceptor layer was to receive photons, while all visual processing is conducted to the optic lobe. With this in mind, estimates of receptor density were therefore made using receptor nucleus counts within the selected retinal region of the inner segment layer. Estimates of nucleus density were modified from the protocol developed for octopus (Young, 1962) as follows: With the MRI retinal topographical map (**Figure 4**), estimates of nuclei density at the visual axis region were based on tangential sections (12 μm thickness). Each sample position represented a rectangular area [100 (W) × 50 (H) μm^2^ – 100 (W) × 200 (H) μm^2^] and within this area all nuclei in the inner segment area between the basal membrane and the retinal plexus layer were counted using the software Fiji. In addition, estimates of cell density were corrected with the equation suggested by Abercrombie (1946), eliminating counting bias particularly of those nuclei partially outside the section plane. Mean of nucleus densities were obtained from 6 to 8 consecutive slices and analyzed using the one-way ANOVA and the general linear model (GLM) for multiple comparisons.

### Immuno-histology of the inner segment layer of squid retinae

The biomarker DiO was used to label lipid membranes of neurons, following a protocol developed for vertebrates (Köbbert et al., 2000). The staining protocol was modified from the basic protocol for the brain slice of mouse (Gan et al., 2000). The fixed squid eyes were isolated and DiO crystals were loaded into both the inner segment and rhabdomeric layers with the glass pipette tip (150 μm diameter) and kept in the light-proof box for 10 days. The two types of the inner segment layers were consecutively cut at a thickness of 25 μm using cryosection. The slices were then incubated with the primary antibody against synapsin (1:50; 3C11 anti SYNORF1, DSHB) in 1% NGS in 0.1M phosphate buffer saline (PBS) overnight at 4°C. The antibody was raised against a GST-synapsin fusion protein of fruit fly in the mouse (obtained from the Developmental Studies Hybridoma Bank developed under the auspices of the NICHD and maintained by Department of Biology, The University of Iowa, Iowa City, IA 52242, USA). After repeatedly rinsing with 0.1M PBS, the Alexa 568-conjugated secondary antibody (1:250; goat anti mouse, a11001, Invitrogen, USA) was applied for 1 h at 4°C. The slices were then embedded in the mounting medium with DAPI (Clear Mount ™ Mounting Solution, Invitrogen, USA) to visualize nuclei. The images were acquired using a confocal microscope (LSM710 META Violet, Zeiss, Germany) and analyzed using the software Fiji (NIH, USA).

### Estimation of resolution and sensitivity of squid eyes

The optical resolution of squid eyes and the absolute sensitivity of an individual photoreceptor were estimated, respectively. The spatial cut-off frequency was determined by the size of the Airy disc (Land, 1981). The size of the Airy disc was suggested to be its half width (*w*). Estimations of the width (*w*) (μm) and the angular image size (θ) (radians) are described by the Equations (1 and 2).

(1) $$\begin{array}{r} w=f\times \frac{\lambda }{A} \end{array}$$

(2) $$\begin{array}{r} \theta =1.22\times \frac{\lambda }{A} \end{array}$$

where *f* is the focal distance; *A* is the diameter of the aperture; λ is the wavelength of light (485 nm for the mid-water species; 500 nm for the coastal species) (Chung and Marshall, 2016).

Visual capabilities were determined by the eye's resolving power *R* and the optical sensitivity *S* developed by Land (1981). Estimates of resolution and sensitivity of squid eyes were adapted from the equations in Land's work. The resolving power (rad^−1^) is defined by Equation (3).

(3) $$\begin{array}{l} R=\frac{f}{2p} \end{array}$$

where *p* is center-to-center receptor separation (μm).

The optical sensitivity *S* (μm^2^ sr) of a simple eye to an extended scene of monochromatic light is defined by Equation (4) as number of photons absorbed per receptor per unit of luminance in the visual scene:

(4) $$\begin{array}{l} S={\left(\frac{\pi }{4}\right)}^{2}A^{2}{\left(\frac{d}{f}\right)}^{2}\left(1-e^{-kl}\right) \end{array}$$

where *d* is the diameter of photoreceptor; *l* is the length of photoreceptor; *k* is the receptive coefficient of photoreceptor.

Chung and Marshall (2014) found that both laser lens tracing and MRI measurement obtain similar results of the focal distance of *Sepioteuthis lessoniana*, therefore, the focal distance, *f*, of this study was determined from the MRI imagery to measure the focal distance from the center of the lens to the hemispherical eyecup using the software Osirix.

## Results

A combination of MRI and histology uncovered several new adaptations of squid eyes: (1) Three types of eyeball deformation. (2) Differentials of screening pigment intensity and movement associating with the light environments. (3) A new form of the dual-layered inner segment retina and the underlying complex neural circuitry. These findings suggest that squid possess complex adaptations in response to vertically diverse aquatic visual environments and modes of life.

### Comparisons between magnetic resonance histology and conventional histology

**Figure 2:**
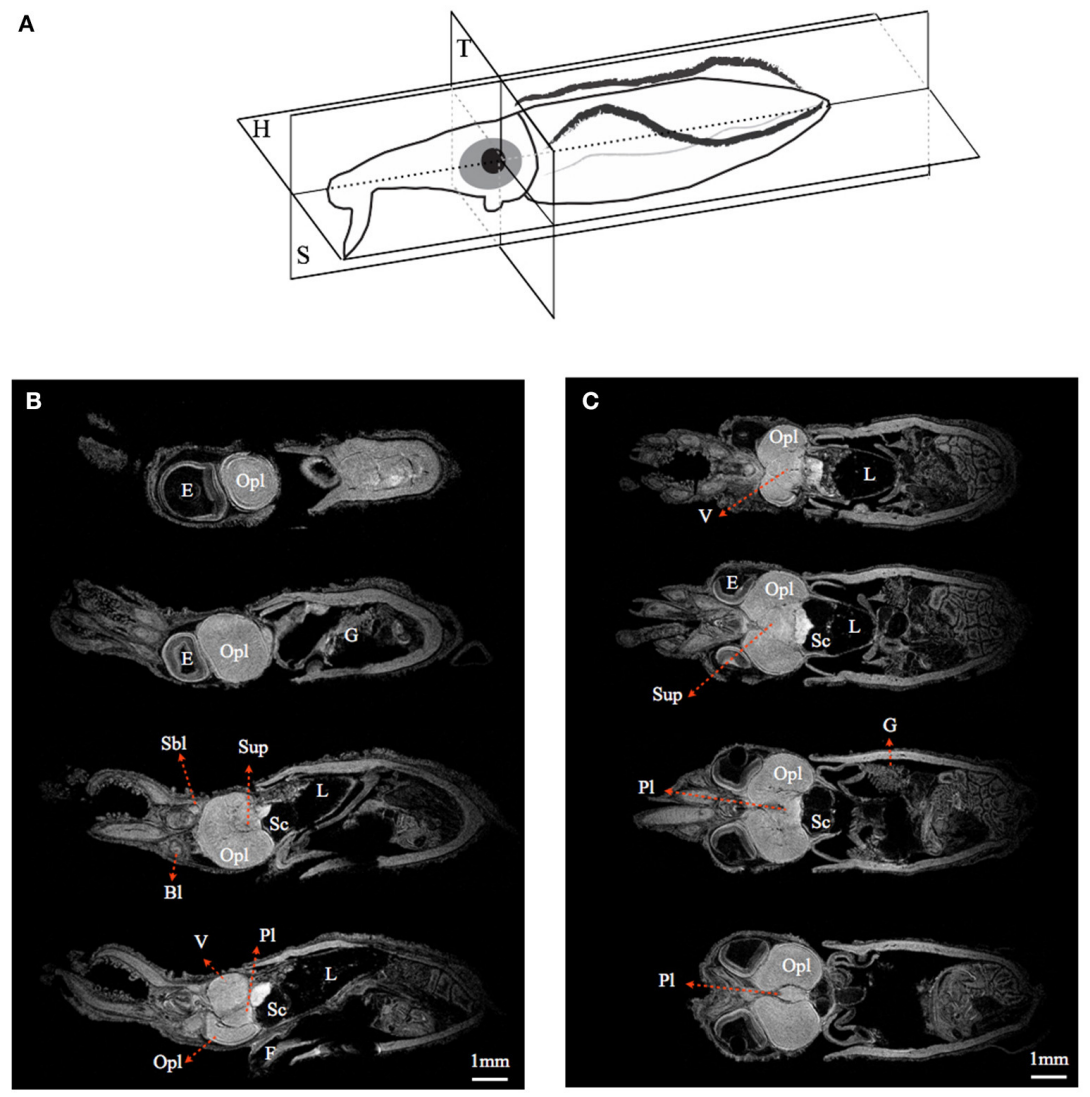
**Magnetic resonance histology of a squid, ***Idiosepius notoides***. (A)** Anatomical planes of squid. Horizontal plane (H). Sagittal plane (S). Transverse plane (T). **(B)** A series of sagittal sections at 200 μm intervals. **(C)** A series of horizontal sections at 200 μm intervals. (E) indicates eye; gill (G), liver (L), ventral lobe (V), brachial lobe (Bl), statocyst (Sc), optic lobe (Opl), pedal lobe (Pl), superior buccal lobe (Sbl), subesophageal mass (Sub), supraesophageal mass (Sup). Voxel resolution: 15μm. Scale bar: 1 mm.

**Figure 3:**
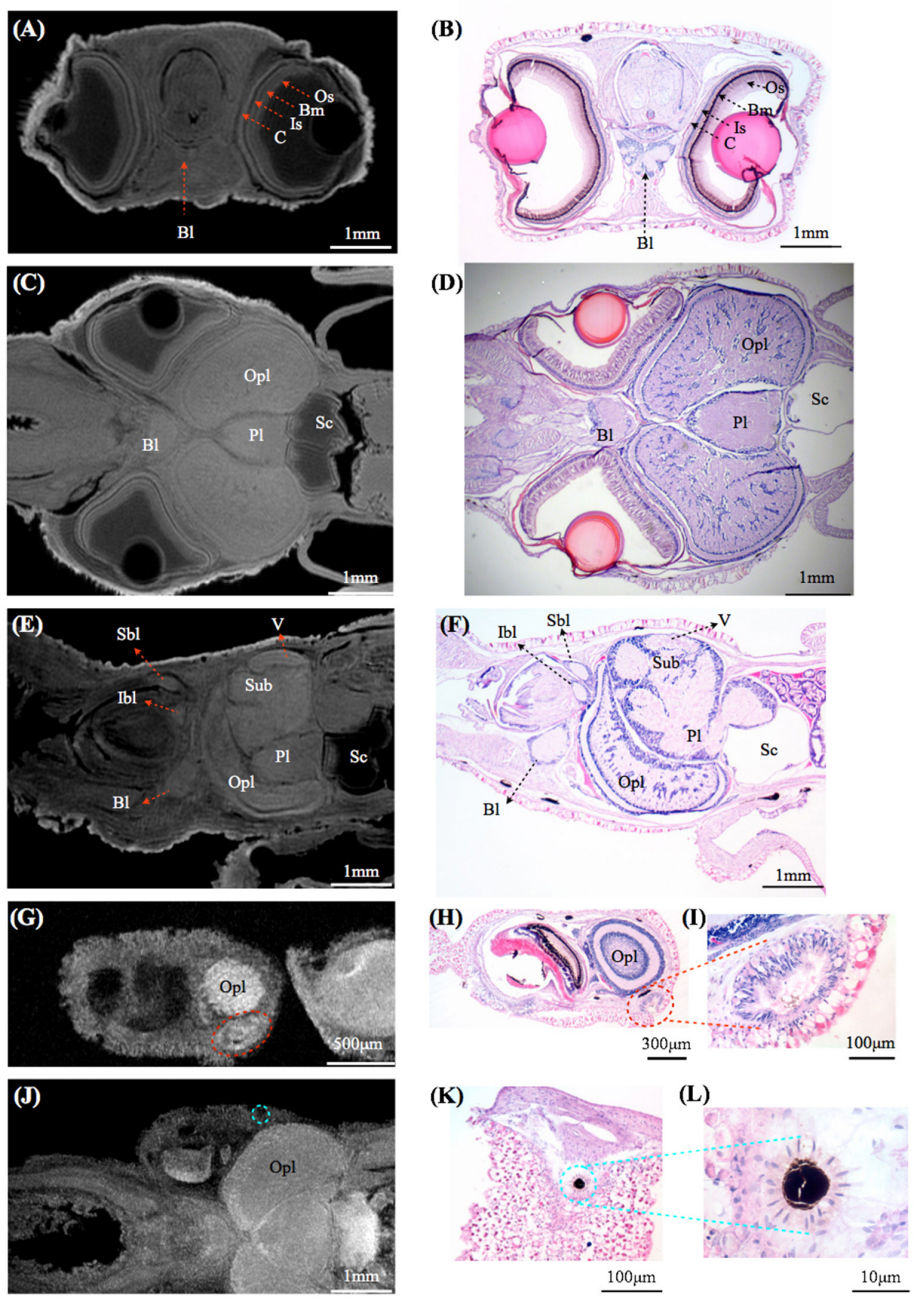
**Comparisons between magnetic resonance histology and conventional histology of a squid, ***Idiosepius notoides***. (A,B)** Transverse sections of squid head. Four different retinal layers can be identified, including the outer segment layer (Os), basal membrane (Bm), inner segment layer (Is), and cartilaginous eye cap (C). **(C,D)** Horizontal sections of squid head. **(E,F)** Sagittal sections of squid head. **(G–I)** Rhinophore. **(J–L)** Chromatophore. (E) indicates eye, gill (G), liver (L), ventral lobe (V), brachial lobe (Bl), statocyst (Sc), inferior buccal lobe (Ibl), optic lobe (Opl), pedal lobe (Pl), superior buccal lobe (Sbl), subesophageal mass (Sub), supraesophageal mass (Sup). The resolution of MRI slice at 16.4T (see Methods) is close to that of standard histology, however in contrast to histological sections that reveal cellular details, no individual cell can be identified in MRI images. Voxel resolution: 9 μm.

By using MRI in combination with classical histology, a detailed brain atlas of *I. notoides* was initiated in three anatomical planes (Figures 2, 3). Neuronal connections inside the central nervous system and the peripheral motor neuron can be traced along individual tracts. Within the optic lobe (OPL), darker regions and boundaries represent the areas containing dense nuclei (i.e., outer granular layer and inner granular layer of the OPL) (Figures 2, 3). Within the retina, four different gray layers in MR images can be discriminated as rhabdom, basal membrane, inner segment, and cartilage layer, respectively (Figure 3). Aside from the detail of retinal structure, neuronal tracks, muscle fibers, brain lobes, other organs can also be discriminated (Figures 2, 3) (Supplementary Videos S1–S4). For this paper, we concentrate only on those features relevant to the visual system comparison.

Comparisons of eyes, optic lobe, and motor center (supraesophageal + subesophageal mass) showed a strong relationship between sensory adaptations and the light conditions where they inhabit (Tables 1, 2). Eye size varied greatly amongst species. In coastal species, the combined eye volume was usually less than half of the head volume. In contrast, pelagic species showed significant eye enlargement relating to increased habitat depth (Tables 1, 2).

**Table 2:** **Volumetric comparisons of squid eyes and lobes**.

**Species**	**Mantle length (mm)**	**Supra-esophageal mass (mm^3^)**	**Sub-esophageal mass (mm^3^)**	**Optic lobes (mm^3^)**	**Central nervous system (mm^3^)**	**Eyes (mm^3^)**	**Head (mm^3^)**	**OPLs/CNS (%)**	**OPLs/Head (%)**	**Eyes/Head (%)**
*I. notoides*	9	0.2634	0.1564	1.79	2.2104	0.8812	3.0916	81.01	57.92	28.50
*S. lessoniana*	19	1.9784	1.6941	13.2	16.8935	11.5146	28.4081	78.26	46.54	40.83
*A. falco*	20	1.2277	0.9854	6.28	8.4973	36.41	44.9073	73.96	13.99	81.08
*P. margaritifera*	19	0.6053	0.4213	2.68	3.709	19.6116	23.3206	72.32	11.50	84.10
*L. reinhardti*	20	0.1691	0.1305	0.71	1.0098	0.2156	1.2254	70.33	57.96	17.59
*S. spirula*	42	8.11	10.61	47.8	67.52	660.2	726.72	71.86	6.58	90.85
*B. abyssicola*	16	0.6298	0.641	2.45	3.7244	10.6218	14.3462	65.88	17.10	74.04

### Non-hemispherical cephalopod eyes

**Figure 4:**
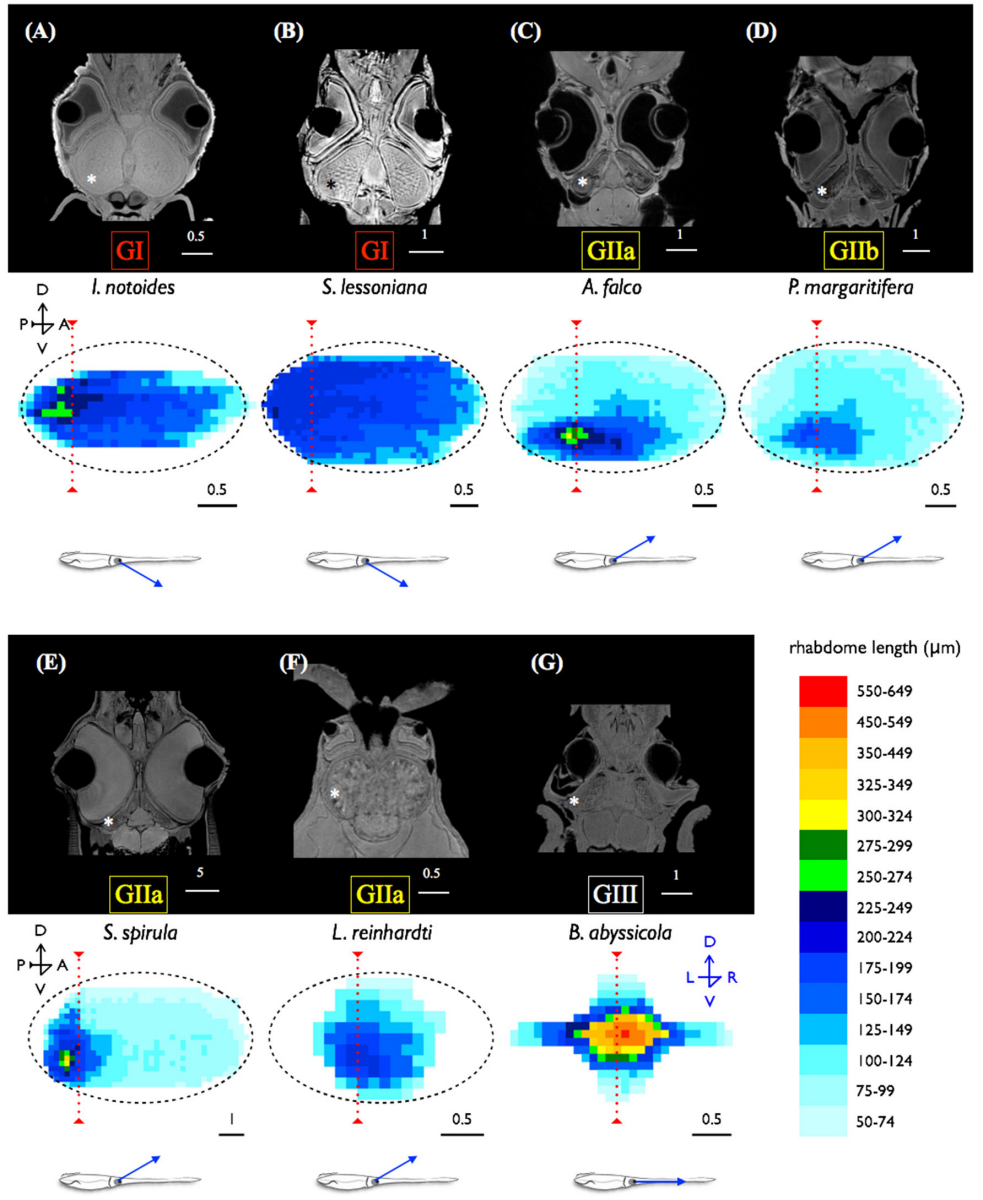
**Morphological variations of squid visual systems and the profile of retinal topography**. Horizontal MRI sections of squid revealed several types of natural eye deformation in squid: non-hemispherical eyes **(A,B,F,G)**; hemispherical eyes **(C–E)**. Histological examinations combined with MRI results demonstrated that the thin (ca. 4 μm) and long rhabdom (>200 μm) patch results in corresponding high visual resolution along the longitudinal section of squid retina (the red dash line). The topography of squid retina revealed that the long and densely-packed receptors and the corresponding visual axis (blue arrow) reflect the visual adaptation to various light environments. ^*^indicates the optic lobe, dorsal (D), ventral (V), anterior (A), posterior (P), left (L), right (R). Scale bar: mm.

**Figure 5:**
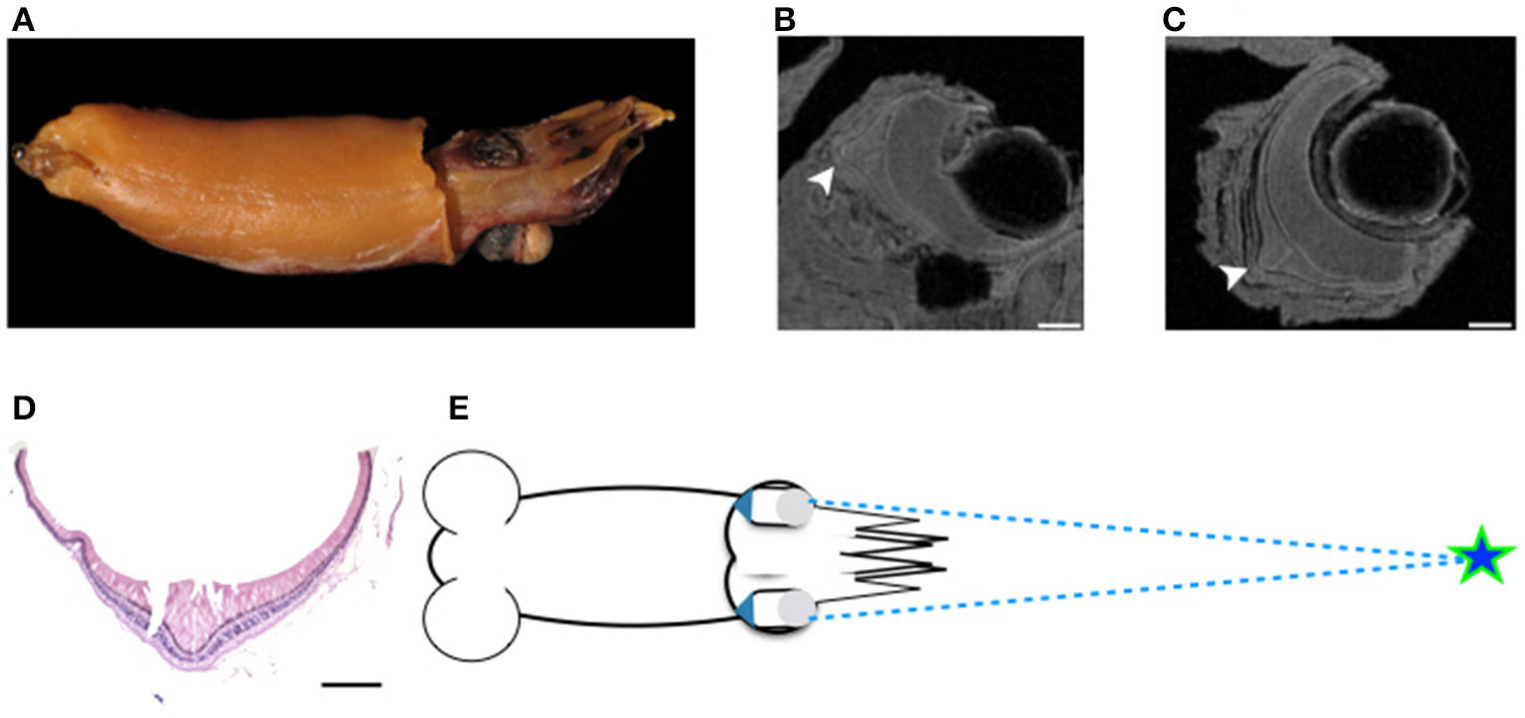
**Tubular eyes and fovea of ***B. abyssicola*** and potential function**. The fovea and tubular eyes could maintain binocular stereopsis and preserve both resolution and sensitivity over a restricted frontal area where is critical for searching prey or mates. **(A–D)** The fovea of *B. abyssicola*. Arrows indicate the fovea. **(D)** A histological section of the fovea which the longest photoreceptor is c.a. 500 μm. **(E)** Illustration of potential function using the tubular eyes in detecting bioluminescent point sources. Scale bar: 500 μm.

**Figure 6:**
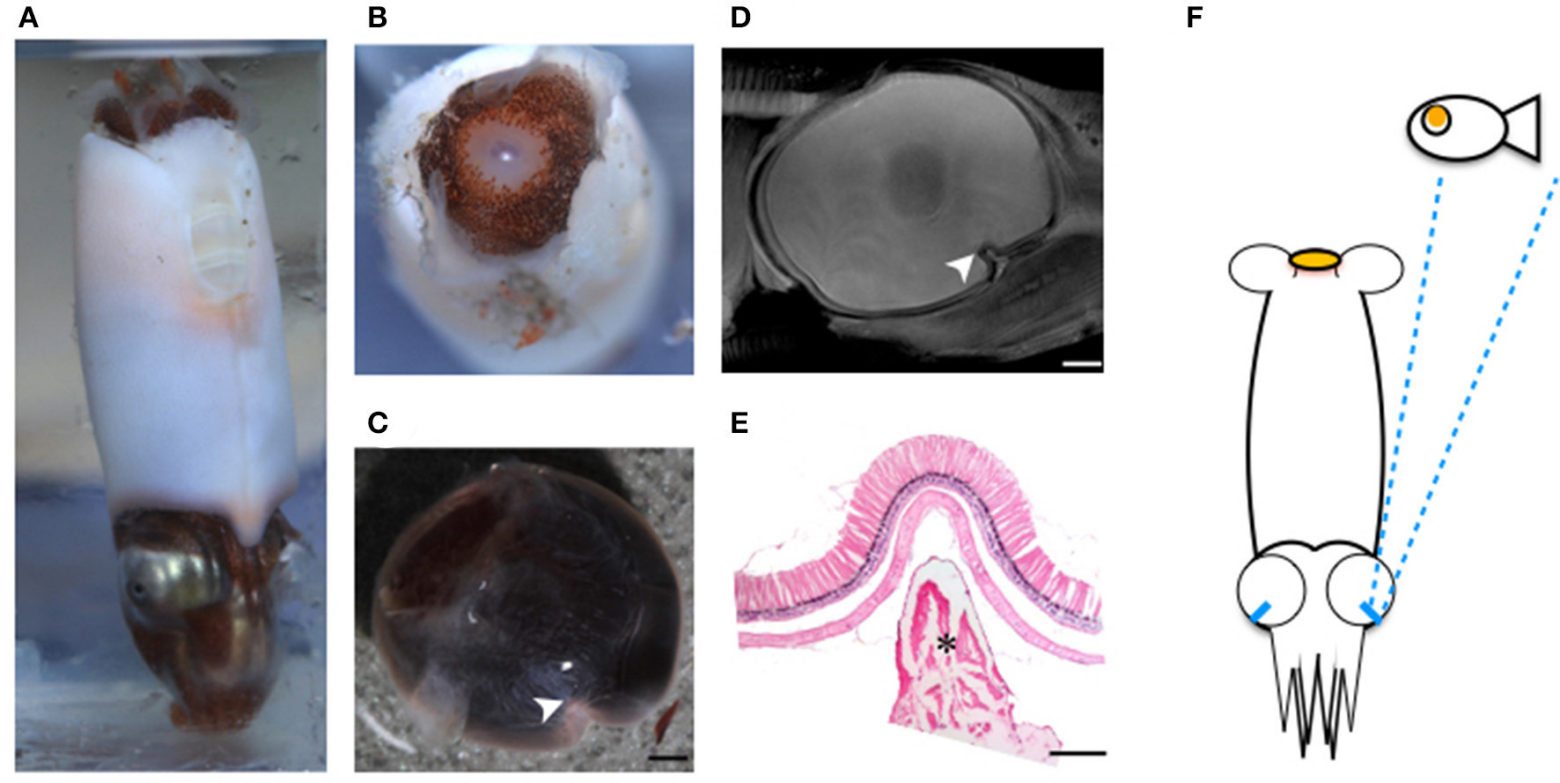
**Regional retinal specialization of ***S. spirula*** and potential function**. With a large photophore emitting light upwards, the pecten-like structure located in the naso-ventral retina combined with its unique head-down posture could be used to detect the object moving overhead. **(A)** The natural head-down posture of live specimen. **(B)** A large photophore at the rear side of mantle. **(C–E)** The retinal ridge. **(C)** The back of the retina is invaded by connective tissue. **(D)** The ridge is located at the naso-ventral side of the eye. **(E)** Histological section of the retinal ridge. (arrow head: the retinal ridge; star: connective tissue). Scale bar: 200 μm. **(F)** Illustration of a potential foraging strategy to search for prey.

MRI images indicate that the three mid-water species, *Abraliopsis falco, Pyroteuthis margaritifera*, and *S. spirula*, possessed a hemispherical eye (Figure 4). In contrast, non-hemispherical eyes were found in the other 4 species (Figure 4) which exhibit three types of regionally modified or deformed retinal structure. There are retinal bumps in two coastal squid species and a fovea-like structure of two deep-sea squid (Figures 4–**6**). The retinal bumps of the two coastal squid result from their enlarged optic lobe (OPL) pressing on the back of retina, forcing much of temporal retina close to the lens (Figures 4A,B). Previous work demonstrated that such eye deformation is not just the result of fixation shrinkage or other sagging artifacts during preparation (Chung and Marshall, 2014). The second type of retinal modification is found in *B. abyssicola*. This species possesses a tubular eye with a foveal pit, in which a patch of very long photoreceptors (>500 μm) is aligned with the central axis of the eye (Figures 4G, 5). The third retinal modification is another invagination or deformation of the retinal hemisphere by a retinal ridge or pecten-like structure, akin to those found in bird eyes (Pettigrew et al., 1990) located in the naso-ventral retina of *S. spirula*, in both juvenile (*n* = 2) and adult stages (*n* = 2) (Figure 6). Anatomy, histology and MRI confirmed that the deformation of *S. spirula* eye is associated with a unique hard “connective” tissue in the orbit invading the back of the retina to form a sharp ridge (Figure 6).

### Dynamic movements of screening pigment granules

**Figure 7:**
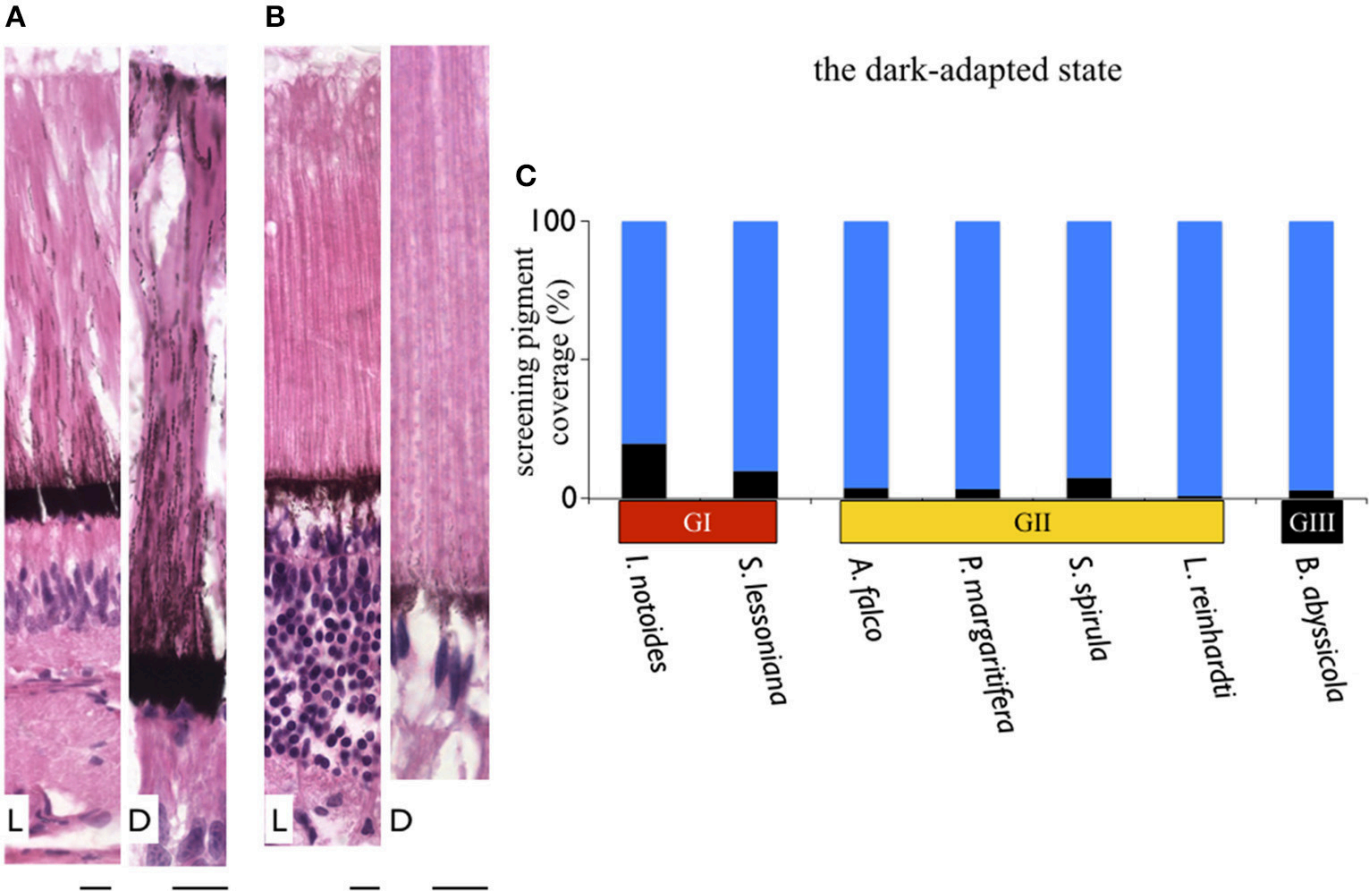
**Variation in thickness of the screening pigment layer in squid**. Differentials of screening pigment density and movement are relating to their living light conditions. In contrast to coastal squid which possess dense and dynamic moving screening pigments to avoid bright sunlight, absence of screening pigment in mid-water squid maximize the amount of light reaching the retina. **(A)** A sample of dynamic movements of screening pigment granules in a coastal squid (*S. lessoniana*). L indicates the light-adapted retina; D as the dark-adapted retina. **(B)** A sample of absence of screening pigment movement in deep-sea squid (*A. falco*). Scale bars: 10 μm. **(C)** Varieties of thickness of the screening pigment layer at the ventral retina of the dark-adapted specimens. Black bars represent the thickness of the screening pigment layer in percentage relative to the length of the rhabdomeric layer.

Dynamic movements of screening pigment granules showed different patterns in light- and dark-adapted conditions (Figures 7A,B). This is in agreement with previous work (Young, 1963). In the dark-adapted state, screening pigment granules were concentrated at the basal membrane, leaving most of the photoreceptor unshielded (Figure 7). The thickness of the dark-adapted screening pigment layer in two coastal squid species covered 10–20% length of the rhabdomic layer. On the contrary, the dark-adapted mesopelagic squid possessed a reduced screening pigment layer where pigments cover only 1–7% of the length of the rhabdomic layer across the retina (Figure 7C).

In the light-adapted state, screening pigment granules of two coastal squid spread out toward the distal end of the outer segment (Figure 7). Screening pigment granules in the dorsal retina dispersed to approximately half the length of the outer segment (~100 μm), leaving the distal region of photoreceptors unshielded by black granules. The ventral retina showed pigments evenly distributed along an entire outer segment (Figure 7). Additionally, a distinctive pigmented band was formed at the tip of the outer segment, known as the outer lamina (~5–10 μm thickness) (Figure 7A). Unlike distinct granule movements seen in coastal squid, screening pigment granules showed no obvious movements in all mid-water species studied here, remaining in the basal region of photoreceptors (Figure 7B).

### Variations of retinal features

Longitudinal sections of *I. notoides* retina (the red dash line in Figure 4A) showed a variance of rhabdom width between 4 and 7 μm. A thin (4 μm) and long rhabdom (200 μm) patch in this species in the dorso-posterior retina results in corresponding high visual resolution, assuming no photoreceptor coupling. This forward-looking area, like the foveal regions in fish, is likely used in predation (Figure 4). Using similar measures of photoreceptor packing density and size, squid retinae from this study can be categorized into three groups: (1) The densely-packed receptors located at dorso-posterior retina in two coastal squid (Figures 4A,B). (2) The densely-packed region located at ventro-posterior retina in 4 mid-water squid (Figures 4C–F). (3) The fovea of the tubular eye in *Bathyteuthis* (Figures 4G, 5).

**Figure 8:**
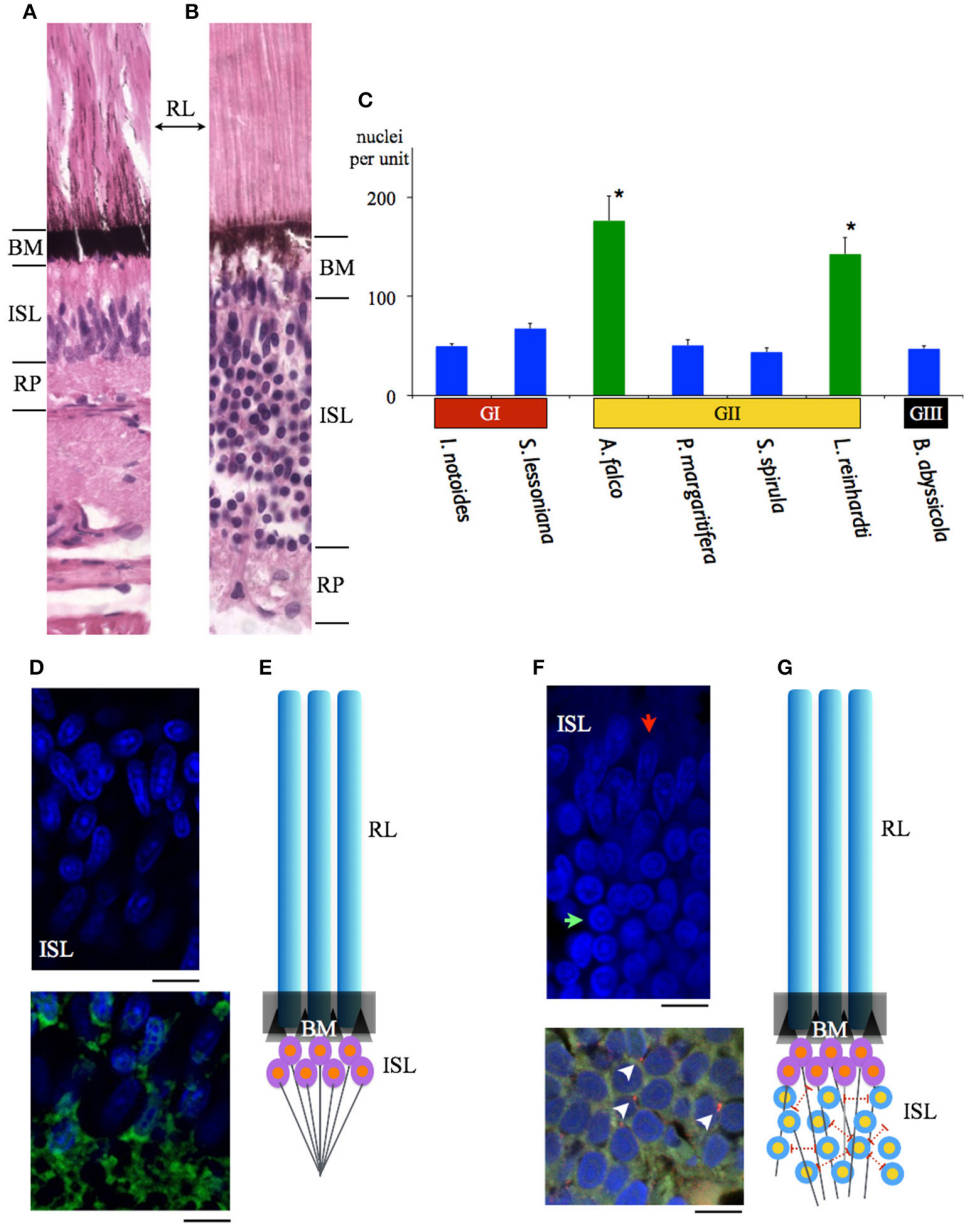
**Comparisons of the maximal density of nuclei amongst 7 squid species inhabiting different depths. (A)** A sample of the previously recognized single inner segment layer of squid retina (*S. lessoniana*). **(B)** A sample of the new form of dual-layered inner segment squid retina (*A. falco*). **(C)** Blue bars indicate the squid which possess the regular single inner segment layer (nuclei density between 44 ± 3.98 – 68 ± 5.12 per unit). The retinal cell density showed no difference. Green bars represent the mid-water vertical migrants (distribution depth between 50 and 1000 m) (nuclei density between 143 ± 16.76 – 176 ± 21.61 per unit). Error bars are ± S.D. ^*^Indicates that these two species possess significant increased cells in the inner segment layer rather than the other five squid. BM indicates basal membrane, inner segment layer (ISL), retinal plexus (RP), rhabdomeric layer (RL). **(D)** Immuno-staining of the regular single inner segment layer of *S. lessoniana*. DAPI (blue florescence) enhanced morpholgical feature of nuclei. DiO (Green fluorescence) showed nerve fibers in the retina plexus layer. **(E)** A schematic drawing of the single-layered inner segment retina. **(F)** Immuno-staining of the dual-layered inner segment retina of *A. falco*. The oval-shape nuclei are below the basal membrane and the round nuclei are located at the lower level of the inner segment layer. (red arrow: oval nucleus; green arrow: round nucleus). Positive synapsin labeling (arrow heads) (red florescence) represents synapse connections. **(G)** A schematic drawing of the dual-layered inner segment retina. Scale bar: 10 μm.

The width of rhabdoms in the densely-packed region among 7 species is 3–4 μm. We further examined the retinal cell density of these regions, and found that 2 mid-water squid species, *A. falco* and *Liocranchia reinhardti*, possess a thickened inner segment layer also exhibiting a 3–5-fold increase in retinal cell nuclei compared to the number of nuclei in the densely-packed retinal region of the other five species (One way ANOVA, *F* = 119.28, *p* < 0.0001) (Figure 8). Most unusually, the nuclei in the thickened inner segment layer of these two species can be grouped into two distinct morphological layers where large numbers of “round” nuclei are placed below a thinner layer of, more commonly observed, “oval” photoreceptor nuclei (Figure 8). As far as we know, this dual-layered inner segment retina has not been observed before in cephalopods.

Immunohistological staining showed an abundance of tubulin in this dual-layered inner segment presumably due to fiber-like supporting or connecting structures. Synapsin is also shown through immunohistochemistry located at junctions between dendrites, and between dendrite and soma (Figure 8). This indicates lateral synaptic connections exist within this dual-layered inner segment retina.

### Optical properties of squid eyes

The optical properties of squid eyes examined here are listed in Table 3. In all species, optical properties were generally similar, resulting the half width of the airy disc (*w*) between 0.5813 and 0.6379 μm. Angular image size and the resolving power per receptor were significantly influenced by the size of aperture, therefore, the tiny *I. notoides* has the lowest angular image size (0.00086 radians) and spatial resolution (92.5 cycles radian^−1^). At the other end of the scale, the largest aperture, found in *S. spirula* rendered the finest angular size (0.00011 radians) and resolution (721 cycles radian^−1^). The optical sensitivity of an individual photoreceptor is between 3.91 and 6.73 μm^2^ sr. Not surprisingly, the highest sensitivity is found in the eye of. *B. abyssicola* (6.73 μm^2^ sr), the deepest living species and one showing a reduced-field tube-eye design associated with an attempt to boost sensitivity (Land, 1981).

**Table 3:** **Optical properties of squid visual system**.

**Species**	**Focal length (μm)**	**Aperture (μm)**	**Receptor diameter (μm)**	**Receptor length (μm)**	**Absorption coefficient**	**Absolute sensitivity**
*I. notoides*	740	580	4	200	0.0067	4.78
*S. lessoniana*	3,800	3,100	4	250	0.0067	5.34
*A. falco*	2,090	1,480	4	240	0.0067	3.96
*P. margaritifera*	3,510	2,930	4	210	0.0067	5.19
*S. spirula*	5,190	4,330	4	170	0.0067	4.67
*L. reinhardti*	5,770	4,560	4	150	0.0067	3.91
*B. abyssicola*	1,836	1,530	4	600	0.0067	6.73
Human^a^ (cone)	16,700	2,000	2	30	0.035	0.02
(Rod)		8,000	20	30	0.035	36.81

a *The optical properties of human from Land (1981)*.

## Discussion

The digitized neural atlas of squid central nervous system started here provides a rapid way to identify the gross anatomy of lobes, complex neural tracks and accurate volumetric estimates of different brain components. Systematic comparisons of volumetric estimates of eyes and visual system reveal that squid eye enlargement is reflected in habitat light conditions. Another important advantage of MRI is to guide or indeed prevent further sectioning of rare deep-sea specimens. In this study, our approach allows a comparative approach that has revealed several new aspects in cephalopod brain and eye structure. Expanded upon in the sections below our two main findings are:

- Adding to previous work on retinal deformation in squid eyes, we add a further two types of modification to cephalopod eye-cup shape. As with the defocused blur of the image for range-finding suggested by Chung and Marshall (2014), these changes in retinal structure appear associated with specific directional visual tasks.
- A combination of MRI and histology demonstrated previously unknown retinal layers. These modifications, are associated with deeper living species and may enhance sensitivity and visual flexibility for rhythmic vertical migration.

### Advantages and challenges of cephalopod brain anatomy using MRI

Comparisons of volumetric estimates of brain lobes with previous work (Maddock and Young, 1987) and results presented here reveal differences in some species (i.e., *B. abyssicola* and *S. spirula*). The animal size of two species used in Maddock and Young's and the current study was different (*B. abyssicola*, 30 vs. 16 mm ML and *S. spirula*, 21 vs. 42 mm ML), therefore, the variations of estimates of lobe volume (i.e., OPLs volume 15.6 mm^3^ vs. 47.8 mm^3^ in *Spirula*) could be due to the differential growth of lobes in different life stages (juvenile vs. adult in these two species). The different segmentation methods between two studies (subsampling sections vs. counting an entire series of sections) might also cause inconsistencies of volumetric estimates. For instance, two similar body size *P. margaritifera* were examined in two studies, however, estimates the OPLs volume showed significant differences as 15 mm^3^ (ML 24 mm) in Maddock and Young (1987) vs. 2.68 mm^3^ (ML 19 mm) in the current study. With classical histological technique in Maddock and Young (1987), the area of a segmentation was measured every 150 μm (every 10th slice for volumetric estimates) in small specimens and one per 600 μm (every 40th slice) for large specimens. In contrast, MRI segmentation in this study included all sequential sections and thus eliminated the problematic alignment of sections. Furthermore, a series of MRI images post-reconstruction allows us to generate 3D images along any stereotaxis plane for analysis, overcoming the methodological constraint of the classical histology of a single angle per specimen.

Although the current squid brain MRI shed new light to anatomical study, a live anatomy squid MRI or functional MRI using this classical neuroscience model animal have got limited progress mainly because of difficulties to keep this creature alive. Unlike successful MRI results using live *Aplysia* and crayfish which have relatively strong tolerance to hypoxia, a small holding chamber (i.e., 35 mm diameter of in our 16.4T scanner) and the associating challenges in oxygen supply and restriction of squid breathing movements during imaging need to be overcome.

### Visual adaptations in different light conditions

A significant problem associated with many visual tasks underwater is maintaining enough sensitivity for various visual tasks in highly variable light environment. Our current study clearly showed that squid have developed visual adaptations rendering some of which are similar those found in fish and some of which appear to be unique to squid. Eye enlargement is a common feature in deep-sea visual predators (Marshall, 1979; de Busserolles et al., 2013). Although enlarged eyes ensure more photons reach to the retina, the eye of the largest pelagic fishes rarely exceeding 90 mm (i.e., swordfish) (Fritsches et al., 2005). On the contrary, the size of large mid-water squid eyes (i.e., *Architeuthis, Dosidicus, Mesonychoteuthis*, and *Octopoteuthis*) certainly exceeds the known largest fish eyes, with eye sizes recorded up to 27 cm (Nilsson et al., 2012).

Recently the giant squid eye, the largest eye on earth, has been suggested to be adapted for the detection of distant point light sources as well as detecting large predators (i.e., sperm whales) illuminated by ambient bioluminescent flashes (Nilsson et al., 2012). Many small mesopelagic squid also possess large eyes relative to their body. For instance, relative eye size given by the eye diameter relative to mantle length was much larger in the firefly squid species studied here (a ratio of ~0.22, 2 species) than those found in lantern fish of similar body size range (eye diameter vs. standard length, between 0.05 and 0.12, 61 species) (de Busserolles et al., 2013). Although enlarged eyes are certainly useful to increase light capture, there are constraints on eye design (especially maximal eye size) such that an extended receptive field rarely increases more than 3 log units of sensitivity (Land, 1981). Using an enlarged eye alone is therefore unlikely to maintain optimal vision during long distance vertical diving or over the day-night cycle. It is for this reason, among others, that many mesopelagic fish in fact migrate up and down in the water column (de Busserolles et al., 2013).

The use of moveable screening pigments at the level of the photoreceptors as well as light-evoked pupillary activities in coastal squid allows a two-stage light attenuation capability and can theoretically tune different retinal regions to different light flux (Young, 1963; Douglas et al., 2005). The two coastal squid studied here possessing the crescent- or w-shaped pupil and its dynamic activities are likely able to offset the vertically uneven luminance from their natural habitat, filtering out large amounts of direct sunlight and improving image contrast in the visual scene as the similar mechanism suggested in cuttlefish by Mäthger et al. (2013).

Deep-sea squid eye design aims to maximize the amount of light reaching the retina at all times. In oegopsid squid, the absence of a corneal membrane and a reduced amount of screening pigment granules discovered in this study minimize these light-attenuated factors (Figure 7). Interestingly, sympatric competitors, fish, have developed a further optical adaptation, the tapetum. This reflective layer located below the retina allows the light that has not been absorbed by photoreceptors to pass through the retina a second time (Warrant and Locket, 2004). Although the reflection of deep-sea squid eye appears light red color, no such reflective layer analogous to the tapetum has been found in any squid studied so far. As the rhabdomeric photoreceptors of squid directly face to the light source and at 200–500 μm are much longer than known fish photoreceptors which are around 10–30 μm (even the extended or multi-bank rods of deep sea fish, which are around 100 μm), having a tapetum is probably not needed in squid.

The fovea of *Bathyteuthis* was previously described only briefly in Chun (1910) and Young (1972). The anatomical measurements provided here indicate this fovea or area is morphologically equivalent to those described in deep-sea fish. Over 50 deep-sea fish species are known to possess an area of higher resolution and this retinal region is presumed to be locked on objects of interest by eye or body movements, in a way similar to the use of the human fovea (Wagner et al., 1998; Warrant and Locket, 2004). The foveal structure (defined as a pit or mound as well as a local increase in photoreceptor density) found in some fish and in *Bathyteuthis* may increase the detection threshold for small bioluminescent object and/or the maintenance of binocular fixation to improve stereopsis (Pumphrey, 1948). Both the tubular eye and fovea in *B. abyssicola* seem to parallel these strategies in deep sea fish. As well as increasing resolution with a fovea-like structure, tubular eyes deliver higher sensitivity over a restricted angular area. So this eye (and those similar in deep-sea fish) provides both increased sensitivity and resolution (Land, 1981).

Aside from the optical adaptations of squid eyes found in the current study and previous investigations (Young, 1963; Sweeney et al., 2007), the molecular basis of spectral tuning in visual pigment has also evolved in response to the dominant spectra of their environment (Chung and Marshall, 2016). Providing both functional and opsin phylogenetic evidence, Chung and Marshall (2016) recently found that squid have evolved depth-dependent spectral tuning, including 4 species in this study, with maximal sensitivity to λmax at 500 nm in coastal squid and blue-shifted λmax of 485 nm in most known mid-water squid.

The optical sensitivity of squid studied here range between 3.91 and 6.73 μm^2^ sr were relatively uniform given the great diversity of depths and habitat, and were similar to the previous estimate of octopus (4.23 μm^2^ sr) in Land (1981). This optically calculated sensitivity is close to the cone cell of pelagic fish such as the blue marlin (1.5–5.6 μm^2^ sr) (Fritsches et al., 2003). Surprisingly, the value of sensitivity of deep-sea squid is far less than the value of deep-sea shrimp (i.e., with *Oplophorus*, 3,300 μm^2^ sr) (Land, 1981). Once optical improvement reaches its physics threshold (i.e., with the multibank retina alone, *Scopelarchus guntheri* is unlikely to increase the sensitivity up to 3 log units), the development of neural summation mechanisms (i.e., spatial or temporal summation) is a more efficient way to increase sensitivity (Warrant and Locket, 2004). For instance, visual sensitivity is improved by a high convergence ratio of photoreceptors to ganglion cells (i.e., the blue marlin, 40:1 at the foveal region) (Collin, 2008). Two mid-water squid species studied here possess a new form of complex retina that contains more and morphologically different nuclei in their dual-layered inner segment layer compared to those which have a regular single retinal layer. As the number of nuclei far exceeds the number of rhabdoms (Φ = 3–4 μm) (Figure 8), we suggest this new form of squid retina could be correlated with the development of a convergent neural circuits potentially providing the dynamic sensitivity adjustment mechanism needed for such a lifestyle.

A series of coleoid cephalopod vertical distribution studies in 1970s revealed that at least four families of mesopelagic squid (Chiroteuthidae, Cranchiidae, Enoploteuthidae, and Histioteuthidae) exhibit extensive diurnal vertical migration between the surface and the bottom level of the mesopelagic realm (c.a. 1,000 m depth) (Clarke and Lu, 1974, 1975; Lu and Clarke, 1975a,b). It is intriguing to note that the new type of the retinal circuit discovered here is associated with squid distribution depths, rather than any phylogenetic relationship. Although two enoploteuthid squid species have a close phylogenetic relationship (Young and Harman, 1998), their retinal design appears to be adapted to their photic ecology or at least depth range. *P. margaritifera* which is predominantly found between 50 and 400 m shows a regular, single-layered retina, while the other species, *A. falco*, possessing the dual-layered inner segment retina inhabit a greater range of depths (Table 1 and Figures 1, 8). Developing a retinal region over which photon catch is pooled (at the expense of spatial resolution) or with increased integration time (at the expense of temporal resolution) has been reported in a great range of taxa (Warrant and Locket, 2004). Thus, the species possessing the dual-layered inner segment retina indicates that they could have signal integration steps. Theoretically, the species with this new form of retinal networks may be more sensitive than those with a single-layered regular squid retina which could only have one convergent process from receptors to the optic lobe (Young, 1974). The neural connections within this type of squid retina are similar to the signal convergence mechanism of photoreceptors to ganglion cells known in deep-sea fish (Wagner et al., 1998).

The proposed signal summation of the dual-layered squid retina theoretically increases sensitivity, a likely adaptation to life in dim environments, but one which comes at the cost of losing spatial resolution. Neural superposition, or at least neural summation is known in the compound eyes of insects to enhance light sensitivity and resolution in nocturnal or crespuscular species, or to drive specific behaviors (Land, 1981; Warrant and Locket, 2004; Agi et al., 2014). The actual function of the dual-layered squid retina needs further evidence to determine and other functions such as polarization e-vector segregation or other signal processing remain possible.

### Unique visual adaptations in squid

Here we documented a second coastal squid, *I. notoides*, has the retinal bump resulting in intentional hyperopic defocus over a half of the frontal scene. Following the squid range-finding mechanism described by Chung and Marshall (2014), the retinal bump and the resulting image blur combined with head bobbing behavior is likely to provide reliable object size and distance information. In contrast to the retinal bump of *S. lessoniana* which disappears at the mature stage (Chung and Marshall, 2014), the retinal bump of the pygmy squid, *I. notoides*, appears in both juvenile and mature stages. This small predator has an adhesive organ to glue itself to the underside of seagrass and waits for prey. Along with this unique sit-and-wait behavior, their head bobbing driven by rhythmic breathing has been clearly recorded (Supplementary Video S5). Visually tracking prey combined with their accurate tentacular strikes despite this defocus suggests that retinal deformation and the resulting new range-finding mechanism might be more common in coastal squid than we expected.

The cranchid squid, *L. reinhardti*, possesses non-hemispherical eyes (Figure 4). Given the eye orientation and the associating receptive visual scene, their vision might be largely restricted in lateral range. It is worth noting that cranchid squid often show significant ontogenetic changes of their morphology in both body shape and eyes (i.e., tubular eyes in larval stage but regular hemispherical eyes in adult) (Young, 1975b; Voss, 1980). Therefore, this non-hemispherical eye may only exist in juvenile stage but return to a regular hemispherical eye shape in adults. The relative requirements for vision at each life stage remain unknown.

The location of the pecten-like structure of *S. spirula* in the naso-ventral retina is unique among coleoids (Figure 6). The gas-chambered shell inside the posterior mantle cavity of *S. spirula*, enables this small cephalopod to float tentacles down in the oceans, resulting in a unique swimming posture of vertical jerky movements (Schmidt, 1922; Brunn, 1943) (Supplementary Video S6). A large photophore at the posterior end of the body may glow continuously, directed upwards (Figure 6). This photophore might work as a light lure to attract prey or as its own torch to emit light for foraging. Theoretically, the glow of its large photophore could result in a strong reflection from animals with tapetum-containing eyes (Warrant and Locket, 2004). *Spirula* might therefore notice the bright reflection from fish eye, enabling a unique foraging strategy. For example, as a point silhouette passes above the animal, the sharp retinal ridge would exaggerate the motion of the moving object (Pumphrey, 1948). In addition, *Spirula* possesses a densely-packed photoreceptor region on ventro-posterior retina as the retinal topography of many mid-water squid (Makino and Miyazaki, 2010), suggesting that this retinal region with its fine optical resolution are important for tentacular strikes (Figure 4E). Without behavioral observation, however, this remains a speculation for future investigation.

## Conclusion

A combination of MRI and histology has discovered several new adaptations of squid eyes, including deformation of modification of eye shape, new photoreceptor arrangements, screening pigment movements, regionally differentiated retina, and a form of complex retina including interneuronal layers previously unknown. Also, a number of previously noted but only briefly described retinal modifications have been examined in more detail, including both pecten-like and fovea-like retinal structures in two deep-sea squid species. These adaptations indicate that squid have developed more complex visual adaptions than previously known in order to survive in various habitats.

## Data accessibility

Data supporting this article is available as an electronic Supplementary Material.

## Ethics statement

The maintenance and experimental protocol used here were covered by animal ethics permit (QBI/223/10/ARC/US AIRFORCE (NF)).

## Author contributions

WC designed the study and prepared the dataset. WC and NM analyzed data and wrote the manuscript.

## Funding

Authors were supported by funding from the Australian Research Council and the Airforce Office of Scientific Research.

### Conflict of interest statement

The authors declare that the research was conducted in the absence of any commercial or financial relationships that could be construed as a potential conflict of interest.
