# Supplementary material for: Complex Visual Adaptations in Squid for Specific Tasks in Different Environments
Source: Front Physiol. 2017 Feb 24;8:105. doi: 10.3389/fphys.2017.00105 (PMC5323406; doi:10.3389/fphys.2017.00105)
Supplement: Supplementary file 7 [file DataSheet1.DOCX]

**Supplementary material**

**Complex visual adaptations in squid for specific tasks in different environments**

Authors: Wen-Sung Chung & N. Justin Marshall

Author for correspondence: Wen-Sung Chung

Email: w.chung1@uq.edu.au

**Supplementary data**

**Figure 1 Sampling locations and methods** A: Moreton Bay, Queensland 2010), B: Coral Sea (RV *Cape Ferguson*, December 2009), C: Peru-Chilean Waters (RV *Sonne*, August 2010), D: Coral Sea (RV *Cape Ferguson*, December 2010), E: Coral Sea (RV *Cape Ferguson*, May 2011).

**Contrast-enhanced magnetic resonance imagery (MRI) of a squid**

Supplementary videos show serial sections from three different planes of *Idiosepius notoides*

**Video S1** Transverse MRI sections of *I. notoides*

**Video S2** Sagittal MRI sections of *I. notoides*

**Video S3** Horizontal MRI sections of *I. notoides*

**Video S4** 3D MRI reconstruction of the head of *I. notoides*

**Video S5** The head bobbing behavior of the pygmy squid, *I. notoides.*

**Unique swimming posture of the ram’s horn squid, *Spirula spirula***

The gas-chambered shell inside the mantle cavity of *S. spirula*, enables this small coleoid to float in the oceans in the range between 100 and 1000 m (Brunn, 1943;Clarke, 1970). The position of the shell in the posterior body requires that the animal usually lives head-down, resulting a unique swimming posture of vertical jerky movements (Schmidt, 1922;Brunn, 1943). Here we showed a video that a live *Spirula* swan in its natural posture (Video S6). Furthermore, a large photophore is located at the posterior end of the body (Main text Figure 6b). The photophore might work as a light lure to attract prey or as its own torch to emit light for foraging (Main text Figure 6f).

**Video S6** *S. spirula* in its natural head-down posture.

References

Brunn, A.F. (1943). The biology of *Spirula spirula* (L.). *Dana Report* 24**,** 1-44.

Clarke, M.R. (1970). Growth and development of *Spirula spirula*. *J Mar Biol Assoc UK* 50**,** 53-64.

Schmidt, J. (1922). Live specimens of *Spirula*. *Nature* 110**,** 788-791.
